# Parietal Mass: Two Case Reports of Rare Cesarean Scar Endometriosis

**DOI:** 10.7759/cureus.6918

**Published:** 2020-02-08

**Authors:** Abdelbassir Ramdani, Kaoutar Rais, Obed Rockson, Badr Serji, Tijani El Harroudi

**Affiliations:** 1 Surgical Oncology, Mohammed VI University Hospital, Regional Oncology Center, Oujda, MAR

**Keywords:** endometriosis, cesarean section, parietal mass, surgery, case report, scar endometriosis, abdominal wall endometriosis

## Abstract

Scar endometriosis is an uncommon type of extra-pelvic endometriosis. However, it should be suspected in any woman of childbearing age complaining of a cyclic, painful nodule in a scar from a previous obstetric or gynecologic procedure, after excluding other differential diagnoses. The treatment of choice is surgical resection. We report two cases of scar endometriosis that appeared in two young ladies after cesarean sections, discovered by a parietal mass near the cesarean scars.

## Introduction

Endometriosis is defined by the presence of ectopic functional endometrial tissue outside of the uterine cavity [1]. It is the main reason for infertility and chronic pain in women of reproductive age. It may be pelvic or extra-pelvic; the most common pelvic sites are the ovaries, posterior cul-de-sac, ligaments of the uterus, pelvic peritoneum, and rectovaginal septum. The major sites for extra-pelvic endometriosis include the lungs, pleura, kidneys, bladder, omentum, bowels, lymph nodes, and abdominal wall [2]. Endometriosis at a scar site can be found after cesarean sections, hysterectomies, amniocenteses, laparoscopic trocar tracts, or perineal episiotomies; the most frequent localization of endometriosis in surgical scars is in the abdominal skin and subcutaneous tissue [3].

We report two cases with cesarean scar endometriosis and refer to a recent review of the literature to discuss all the symptoms and radiology exams that may lead to earlier diagnosis and treatment of abdominal scar endometriosis.

## Case presentation

Case report 1 

**Figure 1:**
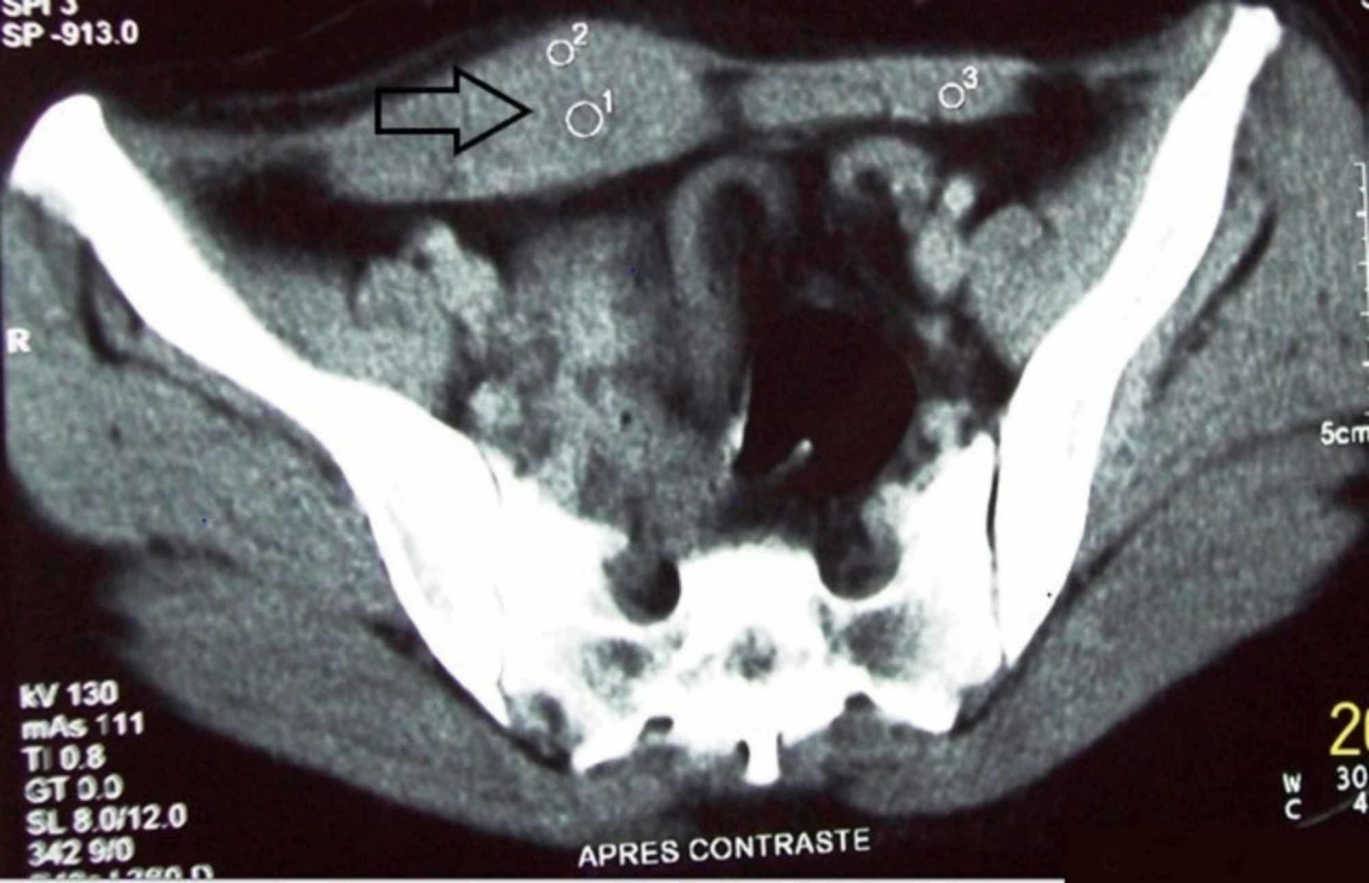
Computed tomography scan showing the endometrioma (black arrow) contained in the rectus abdominis muscle

Case 1 was a 44-year-old female patient, married, and the mother of one child who had, as a surgical antecedent, a cesarean section performed three years prior to presentation. She described a three-month history of the appearance of cyclic hypogastric pain and pain in the right iliac fossa without irradiation and without favoring or sedative factors. The physical examination found a parietal mass at the right iliac fossa measuring 4 cm that was mobile with no inflammatory signs. Ultrasonography showed a subcutaneous right paramedian mass measuring 7 x 3 cm attached to the rectus abdominis muscle that was well-defined, hypoechoic, and heterogeneous with an arteriovenous vascularization on a color Doppler study. A complementary computed tomography (CT) scan with intravenous contrast reported a mass in the rectus abdominis muscle with spontaneously hypodense contents, enhancing heterogeneously, measuring 7 x 3 cm (Figure 1). 

After two negative biopsies with Tru-cut® (Merit Medical Systems, Inc., South Jordan, UT), we decided to perform a transfixing excision of the parietal mass, including a cutaneous flap, and performed an omentoplasty along the path of the biopsy with the repair of the fascial wound by a nonabsorbable plaque. The procedure went well, and the patient experienced no pain following the procedure. The histology results confirmed the diagnosis of parietal endometriosis. The patient experienced no recurrence over the next four years of follow-up.

Case report 2

**Figure 2:**
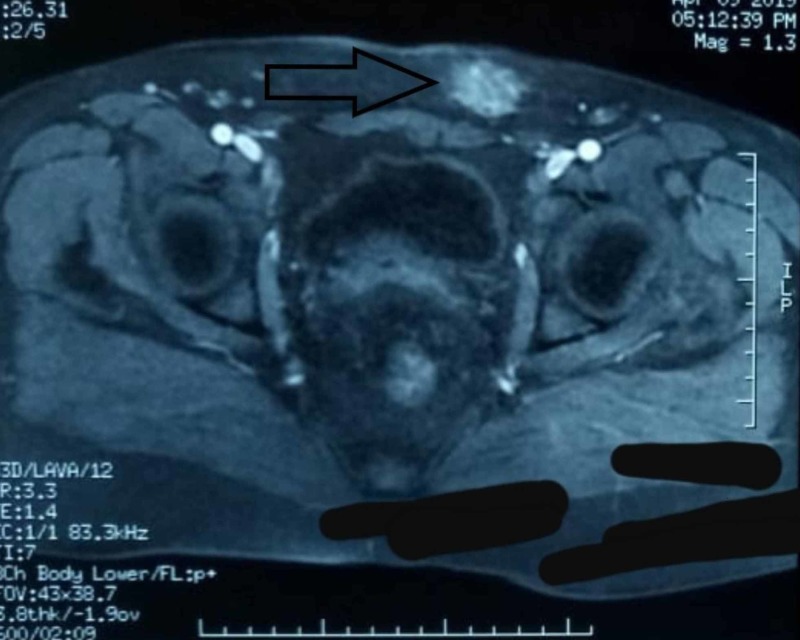
Pelvic magnetic resonance imaging (MRI) scan showing a mass (black arrow) of the left lateral pelvic wall

A 31-year-old female patient, married and the mother of two children, presented with complaints of a painful hypogastric mass next to a cesarean section scar. Her past medical and surgical histories were negative, except that she had undergone two cesarean sections and the mass had appeared two years after her last cesarean section. Physical examination revealed a firm, tender, subcutaneous mass on the left side of the Pfannenstiel incision measuring 2 x 3 cm. A pelvic magnetic resonance imaging (MRI) scan with intravenous contrast revealed evidence of a subcutaneous fat mass of the left lateral pelvic wall, roughly rounded with spiculated contours, measuring 28 x 23 mm, and showing heterogeneous enhancement(Figure 2). 

**Figure 3:**
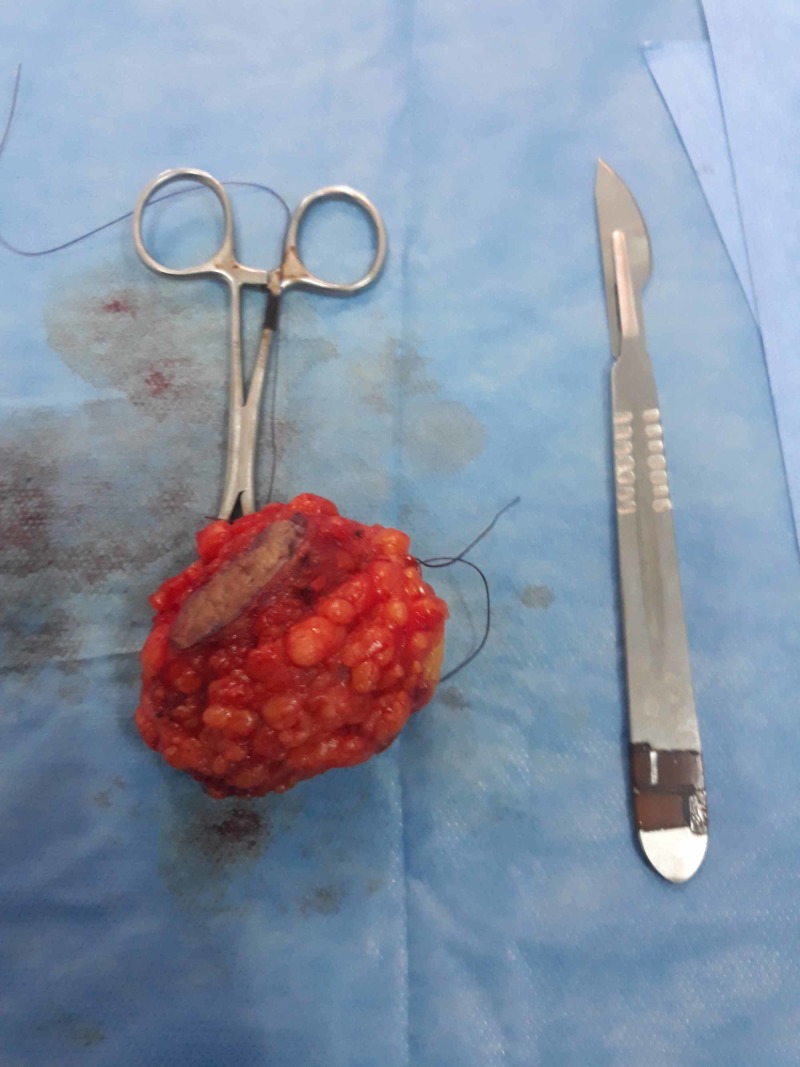
Image showing the resected mass

Under general anesthesia, surgical exploration revealed a mass at the left lower rectus wall, and en bloc excision of the mass was performed (Figure 3). Anatomopathology confirmed the presence of benign fibrous tissue with multiple endometrial glands and stroma, confirming the diagnosis of endometriosis. The patient was seen at regular intervals, and she was symptom-free at the six-month follow-up review.

## Discussion

Abdominal wall endometriosis was first documented by Meyer in 1903 [4]. It is defined as any endometrial tissue found in the superficial peritoneum due to gynecologic surgeries, especially hysterectomy (2%) and cesarean section (< 0.4%) which are the most common provoking factors [5]. However, there are also case reports of spontaneous occurrences discovered in up to 20% of gynecological laparotomies [6]. 

The prevalence of extra pelvic endometriosis is between 9% and 15% in the literature [1]. The most common site of extrapelvic endometriosis is the Pfannenstiel scar which represents 44% of all the 34 cases reported in the 2004 study by Douglas and Rotimi [7]. Paşalega et al. found that the risk of parietal endometriosis in scars was found to be 2.7% after obstetric interventions, 1.5% after gynecologic surgery, and 0.5% after laparoscopic procedures [8].

It is a common gynecologic condition that affects up to 22% women of all age groups: 8-15% in the reproductive age group (most frequently, multipara between 25 and 35 years) and 6% in the premenopausal age group [9]. The pathogenesis of endometriosis in a parietal scar is explained by an iatrogenic direct implantation theory, which suggests that endometrial cells escape through an incision made in the uterus during the surgical procedure and are implanted within the abdominal wound [8-9].

There are two other theories:

- The coelomic metaplasia theory by Meyer suggests that metaplasia of the coelomic epithelium was the origin of endometriosis [4]. This theory possibly explains some rare cases of men who are diagnosed with endometriosis [10].

- The metastatic theory suggests that distant lesions are established by the hematogenous or lymphogenous spread of viable endometrial cells which logically explains the rare endometriotic lesions distant from the uterus [10]. 

The ectopic endometrial cells stimulated by the osteoprotegerin will escape apoptosis, adhere to the underlying peritoneum, generate a new vascular supply, evade the immune surveillance system, and proliferate to build an endometriotic lesion. An increase in the concentration of hepatocyte growth factor and a decrease in the concentration of interferon gamma-inducible protein-10 in the peritoneal fluid of women with endometriosis may stimulate angiogenesis and the development of endometriosis [10]. Some of those functional endometrial cells continue their growth and become malignant, although the overall frequency of malignant transformation from endometriosis is estimated to be up to 1% [11]. The most common type is clear-cell carcinoma with overall survival in five years estimated to be 80% [12]. The resulting endometrial lesions can lead to a chronic inflammatory disorder with increased numbers of activated macrophages and proinflammatory cytokines in the peritoneal fluid that may cause pain and infertility [13].

The manifestations of endometriosis are various in the form of dysmenorrhea, chronic pelvic pain, dyspareunia, and a mass in the abdominal incision during menses [13]. Abdominal wall mass differential diagnoses include desmoid tumor, fibrosis, suture granuloma, fat necrosis, nodular fasciitis, incisional and ventral hernias, hematomas, abscesses, and primary or metastatic malignancies [12]. In general, the history of gynecological or obstetrical surgery may lead us to think about this diagnosis; the interval between the onset of symptoms to past surgery varies from a few months to 10 years [5]. To differentiate between these diagnoses and precisely locate the extent of the lesion, we use imaging tests like ultrasonography which confirm the lesion, even if small, and provides information on its size, location, margins, and internal structure. Ultrasonography can easily differentiate solid from cystic masses. CT or MRI can be used in case the diagnosis is in doubt. MRI is suited best for defining the anatomy of the soft tissue mass and its surrounding structures. The different imaging modalities are nonspecific but useful in determining the extent of disease and assist in the planning of the operative resection [14].

The most common treatment options for scar endometriosis include medical therapy and surgery [15]. Surgical treatment offers the best chance for making a definitive diagnosis and treating cesarean scar endometriosis. The excision should include clear margins of at least 1 cm away from the solid tissue [16]. In addition to this, the abdominal wall endometriosis incorporated into the musculature of the abdominal wall requires en bloc resection of the underlying myofascial elements and the excision site must be sterilized by neomycin solution or 0.9% sodium chloride solution [17-18]. Sometimes, reconstruction of the abdominal wall can be achieved by using a synthetic mesh to prevent a postoperative hernia. The histology of the piece confirms the diagnosis of endometriosis by the presence of endometrial-like glands, spindled endometrial stroma, and hemosiderin deposition either within the macrophages or in the stroma [19]. 

Medical therapy has a low success rate and is associated with adverse effects. Based on a retrospective review of abdominal wall endometriosis made in China between 2003 and 2011, 63 of 229 patients diagnosed with abdominal wall endometriosis took hormonal treatment, such as oral contraceptives, gonadotropin-releasing hormone agonist (GnRHa), danazol, or progesterone. These drugs offer only a temporary alleviation of symptoms that are often followed by recurrence after cessation of drug intake [9]. However, preoperative medical treatment may be useful in reducing the lesion [20]. Instead, used as an adjuvant hormonal therapy after surgical excision, it decreased the recurrence from 42.9% to 11% [8]. Local recurrence is very variable and can occur, especially after an inadequate surgical excision. Bektas et al. found a recurrence rate of 9.1% [1]. In a review of 445 cases, Horton et al. noted a recurrence rate of 4.3% in the range of follow-up evaluations that lasted from one to 18 years [17]. 

Based on implantation theory, there are strategies for preventing the abdominal wall endometriosis, such as lifting the uterus outside of the pelvis before making the uterine incision, using separate needles for the uterine and abdominal closure, removing a functional corpus luteum at the time of the hysterectomy, using high-pressure irrigation, not using a sponge to clean the endometrial cavity, and suturing the uterine incision without endometrium [17]. The strict control of cesarean delivery is strongly recommended [9].

## Conclusions

Despite the rarity of parietal endometriosis pathology, every surgeon must consider it when he is in front of a patient of reproductive age with a history of obstetric or gynecologic surgery and who has suffered from a parietal mass, infertility, or chronic pain. Non-invasive imaging modalities, like ultrasonography, CT scan, and MRI, are helpful to determine the location and extension of the masses, but they are unsatisfactory for confirming a diagnosis. Only the anatomopathological results will allow one to recognize endometriosis. Surgical treatment consists of the total excision of the mass and is still the gold standard treatment with a very low rate of recurrence if done properly. To conclude, awareness of the schedules and all the preventive rules in all gyneco-obstetrical surgery is the most important way to eradicate this disease.
